# Mitochondriolus: assembling mitoribosomes

**DOI:** 10.18632/oncotarget.4646

**Published:** 2015-06-25

**Authors:** Antoni Barrientos

**Affiliations:** Department of Neurology, Department of Biochemistry and Molecular Biology, University of Miami Miller School of Medicine, Miami, USA

The ribosome is an ancient ribonucleoprotein machine universally responsible for catalyzing protein synthesis. Within eukaryotic cells, mitochondria contain their own ribosomes (mitoribosomes), which are highly modified ribosomes of bacterial descent, specialized for the synthesis of a handful of proteins, all essential for the biogenesis of the oxidative phosphorylation system. With high-resolution cryo-EM structures of the mitoribosome becoming recently available, the attention is geared towards understanding the mitoribosome biogenetic process and its compartmentalization.

Spatial compartmentalization is essential for cytoplasmic ribosome biogenesis. Within the nucleus, the membrane-less nucleolus is organized around the chromosomal regions that contain the ribosomal RNAs (rRNA) genes, and is the site of rRNA transcription and processing, and of ribosome assembly. Nascent rRNA transcripts move away from the DNA template and are detected first in the fibrillar and later in the granular nucleolar components. Subsequently, pre-ribosomal particles are released from the nucleolus to undergo sequential maturation in the nucleoplasm and cytoplasm before they acquire translation competence. Within mitochondria, the genome (mtDNA) and proteins involved in mtDNA maintenance, replication and transcription, form a nucleoprotein complex known as the mitochondrial nucleoid. Hundreds of nucleoids are present per cell, each containing 1-5 mtDNA molecules. Recent data has indicated that the assembly of mitochondrial ribosomes occurs near the nucleoid [1], at least partially in bromouridine (BrU)-positive RNA foci that form a compartment known as the mtRNA granule [2–5]. We have renamed this compartment as the mitochondriolus, given that it has features equivalent to the nucleolus [4].

The stages of mitoribosome biogenesis occurring at the nucleoid or at the mitochondriolus are a source of current debate in the field. Formaldehyde cross-linking performed to determine the nucleoid proteins that are in close contact with the mtDNA, led to a model for a layered structure of nucleoids in which replication and transcription occur in the central core, whereas RNA processing and translation may occur in the peripheral region. Affinity purification of known mammalian nucleoid components followed by mass spectrometry identified proteins involved in mtDNA metabolism and additionally many mitoribosome proteins (MRPs) and translation factors, indicating an intimate association among nucleoids, components of the mitochondrial translation machinery and foci containing ribonucleoprotein complexes. Supporting this view, the absence of MPV17L2, a protein required for mitoribosome assembly whose stability depends on mtDNA, induces the trapping of mitoribosome small subunit (mtSSU) proteins in enlarged nucleoids [6]. This has suggested the hypothesis that the mtSSU could be assembled at the nucleoid, enabling the direct transfer of messenger RNA from the nucleoid to the mitoribosome. SILAC pulse-chase experiments followed by sucrose gradient fractionation and proteomics analysis showed that mitochondrial RNA processing enzymes, as well as a subset of MRPs associate with nucleoids or foci located near the nucleoids to initiate RNA processing and ribosome assembly [1]. Although from these data the authors concluded that the first steps in ribosome biosynthesis occurred at the nucleoid, they did not contemplate the possibility the RNA granules co-sedimented with nucleoids in sucrose gradients and therefore the SILAC results cannot exactly discriminate where mitoribosome assembly occurs.

The RNA granules or mitochondrioli contain the core protein component GRSF1, required for mtSSU biogenesis [2-4], RNA processing enzymes, MRPs and ribosomal RNA modifying enzymes and accumulate mtRNAs to regulate their processing, storage, sorting or translation [2, 3]. We approached the question of mitoribosome assembly compartmentalization when we identified the DEAD-box helicase DDX28 [4] as an RNA granule component essential for mitoribosome large subunit (mtLSU) assembly. DDX28 and GRSF1, together with MRPs, had been found to co-purify with tagged mtLSU 16S rRNA methyltransferase MRM3, further supporting that mitoribosome biogenesis could occur at the RNA granule. Recent comprehensive affinity purification studies of GRSF1 and DDX28 followed by mass spectrometry analysis by two independent groups have yielded similar results [4, 5]. In our study, DDX28 was found associated with most MRPs, several previously identified factors involved in the biogenesis of either the mtLSU or the mtSSU, mitochondrial RNA metabolism proteins, and a set of translation factors [4]. These studies also identified a few proteins previously linked to mitochondrial nucleoids.

The overlapping pool of proteins associated with either nucleoids or RNA granules reflects their proximity and the lack of membranous boundaries sealing these compartments. Their functional and physical dynamics is highlighted by the observation that approximately 10-15% of RNA granules co-localize with nucleoids, probably reflecting active transcription sites [2, 3, 7]. A quantitative assessment of these dynamics showed that after a short pulse of 20 min, most BrU-positive RNA foci colocalize within 200 nm of mtDNA, but they become randomly distributed after longer periods or a pulse-chase [7]. Thus, mitoribosome assembly could be initiated near the nucleoids [1, 6], possibly in a co-transcriptional manner as it occurs in bacteria. In a model, newly transcribed rRNAs and/or early mitoribosome assembly intermediates are transferred from the nucleoid periphery to the mitochondriolus, where mitoribosome assembly would be largely completed.

What is the precise composition and structure of the mitochondriolus, how molecular confinement is achieved within this membrane-free compartment, and at which point of maturation mitoribosomal subunits are released from the mitochondriolus and become translationally active are some of the questions that warrant future investigations.
